# Neurodevelopmental Outcomes of Infants Younger Than 90 Days Old Following Enterovirus and Parechovirus Infections of the Central Nervous System

**DOI:** 10.3389/fped.2021.719119

**Published:** 2021-09-28

**Authors:** María de Ceano-Vivas, M. Luz García, Ana Velázquez, Fernando Martín del Valle, Ana Menasalvas, Amaia Cilla, Cristina Epalza, M. Pilar Romero, María Cabrerizo, Cristina Calvo

**Affiliations:** ^1^Pediatric Emergency Department, La Paz University Hospital, Madrid, Spain; ^2^Department of Pediatrics, Severo Ochoa University Hospital, Madrid, Spain; ^3^Department of Pediatrics, La Paz University Hospital, Madrid, Spain; ^4^Department of Pediatrics, Virgen de la Arixaca University Hospital, Murcia, Spain; ^5^Department of Pediatrics, Burgos University Hospital, Burgos, Spain; ^6^Department of Pediatrics, 12 de Octubre University Hospital, Madrid, Spain; ^7^Department of Microbiology, La Paz University Hospital, Madrid, Spain; ^8^National Centre for Microbiology, Instituto de Salud Carlos III, CIBER de Epidemiología y Salud Pública, Madrid, Spain; ^9^Department of Pediatric Infectious Diseases, La Paz University Hospital and La Paz Research Institute (IdiPaz), Madrid, Spain; ^10^Translational Research Network in Pediatric Infectious Diseases (Red de Investigación Traslacional en Infectología Pediátrica), Madrid, Spain

**Keywords:** enterovirus, parechovirus, central nervous system infection, young infant, developmental outcome, ASQ-3 test

## Abstract

Enteroviruses (EVs) and human parechoviruses (HPeVs) are a major cause of central nervous system (CNS) infection in young infants. They have been implicated in neurodevelopmental delay, however limited data are available. The aim of this study is to describe the clinical outcome of young infants and to assess and compare the medium-term neurodevelopment following CNS infections caused by EV and HPeV. A multicentre observational ambispective study was conducted between May 2013 and March 2018. Children under 3 months of age with EV or HPeV CNS infection excluding encephalitis were included. Infants were contacted 1 year after the acute infection and their neurological development was evaluated using the Ages and Stages Questionnaire-3 (ASQ-3). If any area assessed was abnormal during the first round of tests, a second round was completed 6 to 12 months later. Forty-eight young infants with EV and HPeV CNS infection were identified: 33 (68.8%) were positive for EV and 15 (31.3%) for HPeV. At first assessment 14 out of 29 EV (48.3%) and 3 out of 15 HPeV (20%) positive cases presented some developmental concern in the ASQ-3 test. EV-positive infants showed mild and moderate alteration in all domains analyzed and HPeV-positive infants showed mild alterations only in gross and fine motor domains. Significant alterations in communication were observed in EV-positive but not in HPeV-positive infants (31 vs. 0%, *p* = 0.016). At second assessment 4 out of 13 EV-positive patients (30.8%) showed mild to moderate concerns in communication and gross motor function domains and 3 out of 13 (23.1%) showed significant concern in fine motor function. Although CNS infections without associated encephalitis are generally assumed to be benign our study shows that at a median age of 18 months almost half of the EV-infected infants (48.3%) and 20% of HPeV-positive infants presented some developmental concern in the ASQ-3 test. We recommend monitor the neurological development of infants during the first years of life after HPeV CNS infection and especially after EV CNS infection, even in mild cases, for an early intervention and stimulation of psychomotor development if necessary.

## Introduction

Enteroviruses (EVs) and parechoviruses (HPeVs) belong to one of the largest RNA virus families called *Picornaviridae*, that cause infections in both humans and animals (www.picormaviridae.com) and their distribution is widespread around the world. In temperate regions, like in Spain, EV and HPeV infections are most common in spring and summer, in tropical regions infections occur throughout the year (1).

EVs and HPeVs frequently cause infection in children but can infect humans of all age groups. Serological data suggests that over 90% of children have been infected with EV or HPeV by the age of 2 years (2, 3). EV infections occur most frequently in children under 10 years while HPeVs in infants under 2 years (4). EVs and HPeVs may cause a wide variety of symptoms including upper respiratory illness, fever without a source (FWS), febrile rash, gastrointestinal and neurological symptoms. Most infections are silent, mild or subclinical, although severe illness can occur (5, 6). EVs are the most common cause of viral meningitis in children (7) and HPeVs have become the second most common cause of central nervous system (CNS) infection in childhood (8, 9).

In neonates and young children with FWS EV and HPeV are frequently detected (EV 14.1%, HPeV 5.9%) (10), and may be clinically indistinguishable (11, 12); however, some studies including Black et al. (13) have found a significant increase in persistent fever, irritability and feeding problems in these patients with HPeV infection. According to Jong et al. (14), about half of the children younger than 90 days of age admitted due to sepsis-like syndrome had an EV or HPeV infection and Sasidharan (9) detected EV or HPeV in 66% of neonatal cerebrospinal fluid (CSF). Aizawa et al. (15) described the incidence of hospitalization for HPeV-infection as 750/100,000 children under 4 months of age during the 2014 HPeV epidemic in Niigata, Japan; it was possible because the authors conduct virologic evaluation of all young infants at all pediatric care hospitals. HPeV type 3 has been implicated in the most severe infections in this age group (16–18).

Limited data are available regarding the neurodevelopment of infants following CNS infections caused by EVs and HPeVs. The short-term outcome of these infections is usually good, although complications such as seizures and fatality have been described associated with serious conditions such as encephalitis. Infants with HPeV infection without signs of encephalitis seem to have a better clinical outcome than those with a clear diagnosis of encephalitis. Martin del Valle et al. observed a satisfactory medium-term neurological development in infants after HPeV infection (19). HPeV encephalitis is associated with neurodevelopmental sequelae despite the reassuring short-term outcomes of the infection (20). Joseph et al. (21) detected developmental concerns in 14% of HPeV infected infants with documented follow-up.

EV meningitis is a mild disease in short-term, even when EV infection is associated to encephalitis as permanent sequelae are rare, although there are exceptions (7). Jong et al. (14) observed that those young infants with EV and HPeV sepsis-like syndrome who did not require intensive care unit (ICU) admission were not at a higher risk to develop severe neurodevelopmental delay more often than the normal Dutch population. However, there is very little information about the neurodevelopmental outcome in infants following CNS EV infection.

The aim of this study is to describe the clinical characteristics of EV and HPeV infections in young infants (<3 months) and to assess and compare the medium-term neurodevelopment following CNS infections caused by EV and HPeV.

## Materials and Methods

### Study Design, Patients and Procedures

A multicentre observational ambispective study was conducted between May 2013 and March 2018 in collaboration with five Spanish hospitals (La Paz University Hospital, 12 de Octubre University Hospital and Severo Ochoa University Hospital of Madrid; Virgen de la Arixaca University Hospital of Murcia and Burgos University Hospital). The inclusion criteria included the following: children under 3 months of age with EV or HPeV CNS infection excluding encephalitis based on clinical findings, confirmed by RT-PCR in CSF or pleocytosis and RT-PCR-positive in blood, rectal or pharyngeal swabs.

CSF, serum, rectal and pharyngeal swab specimens were collected and analyzed by the hospitals with a commercial RT-PCR for the detection of EV and HPeV (Xpert EV, Cepheid, CA, USA). Positive samples were sent to the Enterovirus Reference Laboratory (National Center for Microbiology) for genotyping. Four RT-nested PCRs specific for species EV-A, B, C and D in 3'-VP1 region and a RT-nested PCR for HPeV in VP3/VP1 region previously published were used for type characterization, followed by sequencing and BLAST analysis (22–24). Bacterial infection was also studied by blood and CSF cultures and ruled out in all cases as a cause of sepsis and CNS infection. Other neurological viruses such as herpes viruses were screened by PCR in CSF. If diarrhea was present, adenoviruses and rotaviruses in stool were analyzed by PCR. Clinical data of infectious episodes were recorded in a specific questionnaire.

Febrile syndrome was defined as an axillary temperature ≥38°C that after an initial examination and laboratory evaluation has no apparent cause. Aseptic meningitis was considered in an infant with fever, irritability, poor feeding, vomits, or bulging fontanelle with pleocytosis (>30 cells/mm^3^ in CSF in neonates and >8 cells/mm^3^ in infants), and with a culture-negative CSF of bacteria. Clinical sepsis was considered in a lethargic infant, with signs related to an alteration of the pediatric assessment triangle (appearance, respiratory and circulatory components).

Infants were contacted by telephone 1 year after the acute infection and their neurological development was evaluated using the Ages and Stages Questionnaire-3 (ASQ-3), which was sent previously by e-mail. The patients were asked to come to the hospital for a physical exam, solve test doubts and sign the informed consent for research participation. If any area assessed was abnormal during the first round of tests, a second round was completed 6 to 12 months later.

The ASQ-3 is a validated screening tool to assess the psychomotor development of infants between 2 and 66 months of age. ASQ-3 is a screening test with age specific questions and analyses five domains of psychomotor functioning: gross motor, fine motor, problem solving, social and communication. The questionnaire includes six closed-ended questions per domain and ten open-ended general questions. Response options for closed-ended questions are: “yes” when the behavior is present (10 points), “sometimes” when the behavior is emerging (5 points), or “not yet” when the behavior is absent (0 points). The questionnaire responses are classified as “significant,” “some” or “no” developmental concern according to ASQ defined subscale cut-off scores (25–27).

Clinical, epidemiological, ASQ-3 test results and laboratory features were described and compared between infants with EV or HPeV infection of the CNS.

This study was approved by the Ethical Committee of clinical Research from the University Hospital La Paz, Madrid, Spain (PI-3092) and from the Instituto de Salud Carlos III (PI18CIII-00030).

### Statistical Analysis

Clinical, laboratory and ASQ-3 test results of HPeV-positive patients were compared with those of the children infected with EVs. Qualitative data were expressed as absolute and relative frequencies and quantitative data as median and interquartile range (IQR). Categorical variables were compared using chi-square and Fisher's exact test, and continuous variables with Student's *t*-test or non-parametric tests as appropriate. A two-tailed value of *p* < 0.05 was considered statistically significant. All analyses were performed using the Statistical Package for the Social Sciences, version 21.0 (IBM Corp., CA, USA).

## Results

Forty-eight young infants with EV and HPeV CNS infection were identified: 33 infants (68.8%) were positive for EV and 15 infants (31.3%) for HPeV. Twenty-five patients were girls (45.8%); no statistical difference between EV and HPeV groups in gender was found. The median age was 21 days (IQR: 13–32), and 20/33 cases were EV-positive (60.6%) while 11/15 cases were HPeV-positive (73.3%) infants under 1 month of age. Four cases (12.1%) in EV group were late preterm (one patient was 35 weeks gestational age and three were 36 weeks). No preterm neonates were present in the HPeV group.

**Clinical characteristics** of infants with EV and HPeV infections are described in Table 1. None of the included patients presented myocarditis. Three HPeV-infected cases had liver dysfunction. The neurological examination at discharge was normal in all patients. Five patients (12.5%) underwent neuroimaging tests as per medical criteria: four had marked irritability and one presented apneas; three belonged to the EV group (one CT, one MRI and two brain ultrasound), and two were from the HPeV group (brain ultrasound). Imaging results were all normal. EEG was performed in 4 cases and all were also normal.

**Table 1:** Clinical and laboratory findings of infants with EV and HPeV infections included in this study.

	**EV-infected patients (*n* = 33)**	**HPeV-infected patients (*n* = 15)**	***p***
**Season**
Winter	9 (27.3%)	1 (6.7%)	NA
Spring	**13 (39.4%)**	**5 (33.3%)**	NA
Summer	3 (9.1%)	**7 (46.7%)**	NA
Autumn	8 (24.2%)	2 (13.3%)	NA
**Clinical features**
Male	15 (45.4%)	8 (53.3%)	NS 0.757
Age (days)	24 ± 14.65	23.6 ± 15.12	NS 0.921
Prematurity	4 (12.1%)	0 (0%)	NS 0.159
Fever	32 (97%)	15 (100%)	NS 1
Irritability	9 (27.3%)	7 (46.6%)	NS 0.182
Upper respiratory infection	9 (27.3%)	0 (0%)	**0.041**
Apnea	2 (6.1%)	0 (0%)	NS 1
Bronchiolitis	1 (3%)	1 (6.7%)	NS 0.532
Gastroenteritis	2 (6.1%)	4 (26.7%	NS 0.067
Rash	12 (36.4%)	2 (13.3%)	NS 0.171
Breastfeeding	24 (72.7%)	15 (100%)	NS 0.157
**Diagnosis**
Febrile syndrome	15 (45.5%)	7 (46.7%)	NS
Clinical sepsis	4 (12.1%)	8 (53.3%)	**0.004**
Meningitis	14 (42.4%)	0 (0%)	**0.002**
Antibiotic therapy	32 (97%)	15 (100%)	NS 1
Pediatric/neonatal ICU admission	5 (15.2%)	8 (53.3%)	**0.012**
Respiratory support	3 (9%)	2 (13.3%)	NS 0.642
**Laboratory findings**
Leucocytes (cells/mm^3^)	8,775 ± 3,651	6,832 ± 2,190	NS 0.063
Serun CRP (mg/L)	9.74 ± 12.46	5.72 ± 6.42	NS 0.247
Procalcitonin (mg/mL)	0.19 ± 0.12	0.24 ± 0.14	NS 0.313
Pleocytosis	15/30 (50%)	0/15 (0%)	**0.001**
CSF cells/mm^3^	89 ± 140.7	3.2 ± 4.7	**0.024**
CSF proteins (mg/dL)	91.55 ± 57.4	51.74 ± 20.1	**0.016**
CSF glucose (mg/dL)	49.5 ± 10.2	54.5 ± 6.4	NS 0.102
Liver dysfunction	0 (0%)	3 (20%)	**0.026**
CSF RT-PCR positive	31 (94%)	15 (100%)	
Pharyngeal swab positive	10/14 (71.4%)	0	
Rectal swab positive	12/13 (92.3%)	0	
Blood RT-PCR positive	3/3 (100%)	0	
Short-term sequelae	0	0	

*Bold values highlight significant data*.

**Significant differences in clinical diagnosis** were observed between both groups. Clinical sepsis was more frequent in the HPeV group (53.3 vs. 12.1%; *p* = 0.04) whereas meningitis was prevalent in EV-positive infants (42 vs. 0%; *p* = 0.002). Pleocytosis in CSF was absent in HPeV infections and present in 50% of EV-positive patients (*p* = 0.001). Upper respiratory infection was associated only with the EV group. Admission to ICU was more frequent in HPeV-infected infants (53.3 vs. 15.2%; *p* = 0.012). The short-term outcome was favorable in all cases.

The **microbiological diagnosis** revealed that HPeVs were identified in the CSF samples of all positive-cases (100%) and EVs in 94% of CSF samples of positive-cases. EV was detected in 10/14 (71%) of pharyngeal swabs and in 12/13 (92%) of rectal swab samples; no rectal or pharyngeal swabs were collected in HPeV-infected patients. Genotyping was reached in 16/33 (48.5%) of EV-positive samples and in 14/15 (93.3%) of HPeV-positive samples. Specific types identified are listed in Table 2. Coinfections were present in six cases (12.5%), five (15.2%) in the EV group (4 urine infections by *Klebsiella, E. coli, S. agalactiae* and 1 adenovirus in feces) and one (6.7%) in the HPeV group (RSV infection).

**Table 2:** EV and HPeV types identified in the samples from patients included in this study.

**EV type**	**Number of cases**
Echovirus 3	1
Echovirus 5	6
Echovirus 9	1
Echovirus 18	2
Echovirus 20	1
Echovirus 25	1
Echovirus 30	1
Coxsackievirus B3	1
Coxsackievirus B1	1
EV-A71	1
Untyped	17
Total EV	**33**
**HPeV type**
HPeV-3	14
Untyped	1
Total HPeV	**15**

*EV, enterovirus; HPeV, parechovirus*.

*Bold values means the total amount of the virus type*.

Almost all cases received antibiotic therapy (47/48, 97.9%). Ampicillin and cefotaxime were the most frequent combination given to the patients. Thirteen cases required ICU admission, five in EV-positive and eight in HPeV-positive infants (15.2 vs. 53.3%, *p* = 0.012). Five infants needed respiratory support, three in EV and two in HPeV-infected patients.

The first **interview and the ASQ-3 test** were completed at least 1 year after infection in 44 patients (29 EV and 15 HPeV-positive cases) at a median age of 18 months (IQR: 14–24). Results of neurological outcome at first assessment are shown in Table 3. Fourteen out of 29 EV (48.3%) and 3 out of 15 HPeV (20%) positive cases presented some developmental concern in the ASQ-3 test. EV-positive infants showed mild and moderate alteration in all domains analyzed in ASQ-3 and HPeV-positive infants showed mild alterations only in gross and fine motor domains. Significant alterations in communication were observed in EV-positive but not in HPeV-positive infants (31 vs. 0%, *p* = 0.016). Gross and fine motor alterations were observed in both the EV and HPeV-positive groups. Problem solving and personal-social domains were altered only in EV-positive infants. Out of the four late preterm infants with EV CNS infection, three showed alterations in ASQ-3 test; communication domain was altered in three, problem solving and fine motor function in two, and gross motor function in one. No significant clinical differences have been found between EV-infection cases with and without neurodevelopment delay.

**Table 3:** First assessment of neurodevelopment in medium-term with the ASQ-3 test.

**Altered domain**	**EV-infected patients (*n* = 29)**	**HPeV-infected patients (*n* = 15)**	***p***
>1 domain altered	14/29 (48.7%)	3/15 (20%)	NS 0.068
Communication	9/29 (31%)	0/15 (0%)	**0.016**
Gross motor	8/29 (27.6%)	2/15 (13.3%)	NS 0.285
Fine motor	5/29 (17.2%)	2/15 (13.3%)	NS 0.737
Problem solving	5/29 (17.2%)	0/15 (0%)	NS 0.088
Personal-social	6/29 (20.7%)	0/15 (0%)	NS 0.058

*Bold values highlight significant data*.

**Second assessment** was requested in 14 of EV-positive patients but this was possible only in 13 cases, at least 6 months after the first assessment (Table 4). The median age at the second test of the 13 EV-positive patients was 24 months (IQR: 18–32). Four out of these 13 patients (30.8%) showed mild to moderate concerns in communication and gross motor function domains. Significant developmental concern was also found in fine motor function (3 patients, 23.1%) and in problem solving and personal-social domain (1 patient, 7.7%). A second assessment was performed in one out of the three late preterm patients, in whom communication domain remained altered. A second test was requested for three HPeV-positive infants, however only one infant answered it and showed a mild alteration in the fine motor domain that improved during the second assessment.

**Table 4:** Second assessment of neurodevelopment in medium-term with the ASQ-3 test.

**Altered Domain**	**EV (*n* = 13)**	**HPeV (*n* = 1)**
>1 domain altered	6/13 (46.15%)	0/1 (0%)
Communication	4/13 (30.8%)	0/1 (0%)
Gross motor	4/13 (30.8%)	0/1 (0%)
Fine motor	3/13 (23.1%)	0/1 (0%)
Problem solving	1/13 (7.7%)	0/1 (0%)
Personal-social	1/13 (7.7%)	0/1 (0%)

## Discussion

There are very few studies evaluating the medium-term neurodevelopmental outcomes of infants with HPeV infection of the CNS such as meningitis and encephalitis, and even fewer with infections caused by EV. We conducted this study in order to assess and compare the medium-term neurodevelopment in young infants following EV and HPeV CNS infection excluding encephalitis.

We performed a multicentre observational ambispective study for nearly a period of 5 years in Spain. We included 48 young infants <3 months of age with EV or HPeV infection of the CNS excluding encephalitis. However, as encephalitis was excluded based on clinical criteria, since MRI and EEG has not been routinely performed, mild cases could have been included in this series. In accordance with previous reports, neurological EV infection was more frequent than HPeV infection in our findings (1, 4, 28). In both study groups, the most frequent diagnosis was febrile syndrome, accounting for almost half of the cases. However, meningitis was more prevalent in infants with EV infections and clinical sepsis in HPeV infections as described by previous authors (14, 17, 29). We further observed that the admission to the ICU and clinical sepsis were significantly more frequent in HPeV-infected infants, as previously reported by our research group (1, 17). EV and HPeV infections are becoming better known to pediatricians, and although PCR is not available in all hospitals, it should be generalized.

Although CNS infections without associated encephalitis are generally assumed to be benign (8, 30), only few data have been published about the neurodevelopment following neurological infections caused by EV and HPeV. Even though the short-term outcome of these infections is usually good, our study shows that at a median age of 18 months almost half of the EV-infected infants (48.3%) and 20% of HPeV-positive infants presented some developmental concern in the ASQ-3 test higher than expected in the European child population, which is between 5 and 10% (31). We observed that EV-positive infants showed mild and moderate alteration in all domains analyzed in the ASQ-3 while HPeV-positive infants showed mild alterations only in gross and fine motor domains. This observation suggests that EV infection in young children is not as benign as previously thought. In the subsequent follow-up 6 months later, at a median age of 24 months, 30.8% of infants in the EV group showed mild to moderate concern in communication and gross motor function domain. In the only HPeV-positive case studied at second assessment, fine motor function domain normalized.

As previously mentioned, 20% of HPeV-infected infants showed mild alterations only in the gross and fine motor domains in the first assessment in our series, whereas communication, problem solving, and personal-social domains were normal. In the second assessment, when possible, fine motor function was recovered. Our findings are consistent with those described by Hinsbergh et al. since children showed a suspect gross motor function delay at 6 months that was normalized during 24 months follow-up, but no longitudinal association was found between HPeV CNS infection and gross motor function (32). Hinsbergh et al. (33) conducted a systematic review and meta-analysis of neurological and neurodevelopmental outcomes in newborns and young children after HPeV CNS infections, including encephalitis. The authors found an increasing proportion of children with neurological sequelae over time, in 5% of cases during short-term follow-up, and increased to 27% during long-term follow-up. Neurodevelopmental delay was suspected in at least 9% during long-term follow-up. The wide range of sequelae observed may be due to the high methodological heterogeneity of the included studies related to selection and follow-up criteria. These results should be therefore interpreted with caution. The meta-analysis included a variable proportion of preterm infants and cases of encephalitis that are not present in our series. Despite the methodological differences, the results were relatively similar to ours with a significant percentage of children with neurodevelopmental concern (27 and 20%, respectively). In a previous study from our group by Martin Del Valle et al., a satisfactory medium-term neurological development was observed in infants following HPeV infection, although mild alterations in gross and fine motor domains were present (19). The differences between study outcomes could be due to variations including the pathogenicity of HPeV, maternal immunity, age of developing infection or differences in host response to the virus (30).

There is very little information about the evolution of EV infections in neurodevelopment in infants. Our study showed that almost half EV-infected cases presented some developmental concern in the ASQ-3 test at the median age of 18 months. They demonstrated mild and moderate alterations in all domains analyzed. Significant alterations in the communication domain were observed in one third of the cases. Gross and fine motor delay, and alterations in problem solving and personal-social domains were found additionally in an important proportion of cases. Second assessment was performed in 13 EV-infected infants (median age of 24 months); one third of the infants also showed mild to moderate concern in the communication and gross motor function domain. These findings are consistent with those published by Wilfert et al. (34), a case control study were receptive language function was significantly altered in infants with EV meningitis compared to controls. In 1975, Sells et al. (35) conducted a follow-up-controlled study of 19 children with documented EV CNS infection (CSF-positive by cell culture) and concluded that children whose illness occurred during the first year of life have significantly smaller mean head circumferences, lower intelligence quotient and depressed language and speech skills compared to controls. Current diagnostic techniques for EV detection (RT-PCR) are more sensitive than those used in the seventies (cell culture), therefore the studies may not be comparable. To the best of our knowledge, our study is the first to investigate neurodevelopmental outcomes of infants following viral infections caused by EV and HPeV. It is worth highlighting that the immature brain of infants under 1 year of age and especially those under 3 months may be more susceptible to CNS damage than infants over 1 year (9).

Prematurity is an independent risk factor of motor disability and other developmental problems (36). Van Dokkum et al. (37) compared the neurodevelopment of 1,247 preterm and 488 full term children and found motor function delay in preterm children especially in those with lower gestational age. However, Woythaler et al. (38) reported that late preterm infants had worse developmental outcomes than term infants with a two-times higher risk for psychomotor delay and intellectual disability at 2 years of age. Gutierrez-Cruz et al. (39) also detected significantly lower language scores in late preterm children. In our series, three infants in the EV group were late preterm and had some developmental concern in the ASQ-3 test, which could have influenced our results.

Our study has several limitations. Firstly, the small sample size. Secondly, not all patients with initial neurodevelopmental concern could undergo a second evaluation. Only a few children had neuroimaging tests at the time of infection, because of the good short-term outcome. The ASQ-3 test was not performed in a control group. The main strengths of our study are its multicentric design, as well as the homogeneity of the included patients; all children were in the same age range, with similar levels of disease severity and confirmed microbiological diagnosis. Despite the limitations, our results suggest that CNS infections caused by HPeV and EV may have an impact on the medium-term neurological development in very young infants, even in mild cases.

As far as we know, this is the largest study that describes the neurodevelopmental evolution of EV CNS infection in young infants excluding encephalitis and shows the presence of neurodevelopmental delay in a significantly number of cases. Therefore, we recommend that clinicians monitor the neurological development of infants during the first years of life after HPeV CNS infection and especially after EV CNS infection, even in mild cases, for an early intervention and stimulation of psychomotor development. The ASQ-3 test is an easy and useful tool to screen post-viral neurological development. Additional prospective studies with a larger study population and longer follow-up are necessary to confirm our results.

## Data Availability Statement

The raw data supporting the conclusions of this article will be made available by the authors, without undue reservation.

## Ethics Statement

The studies involving human participants were reviewed and approved by Ethical Committee of clinical Research from the University Hospital La Paz, Madrid, Spain and from the Instituto de Salud Carlos III. Written informed consent to participate in this study was provided by the participants' legal guardian/next of kin.

## Author Contributions

CC, MG, and MC-V contributed to conception and design of the study. MC-V and AV organized the database. CC, MG, and MC-V performed the statistical analysis. MC-V and CC wrote the manuscript. All authors contributed to collection of data, manuscript revision, read, and approved the submitted version.

## Funding

This study was partially supported by grant from Instituto de Salud Carlos III (number PI18CIII/00017).

## Conflict of Interest

The authors declare that the research was conducted in the absence of any commercial or financial relationships that could be construed as a potential conflict of interest.

## Publisher's Note

All claims expressed in this article are solely those of the authors and do not necessarily represent those of their affiliated organizations, or those of the publisher, the editors and the reviewers. Any product that may be evaluated in this article, or claim that may be made by its manufacturer, is not guaranteed or endorsed by the publisher.
